# Supplementary figures and images for: Reduced legacy precipitation decreases microbial community growth efficiency and alters soil organic carbon in a California grassland
Source: Microbiome. 2026 Apr 11;14:150. doi: 10.1186/s40168-026-02395-9 (PMC13188737; doi:10.1186/s40168-026-02395-9)

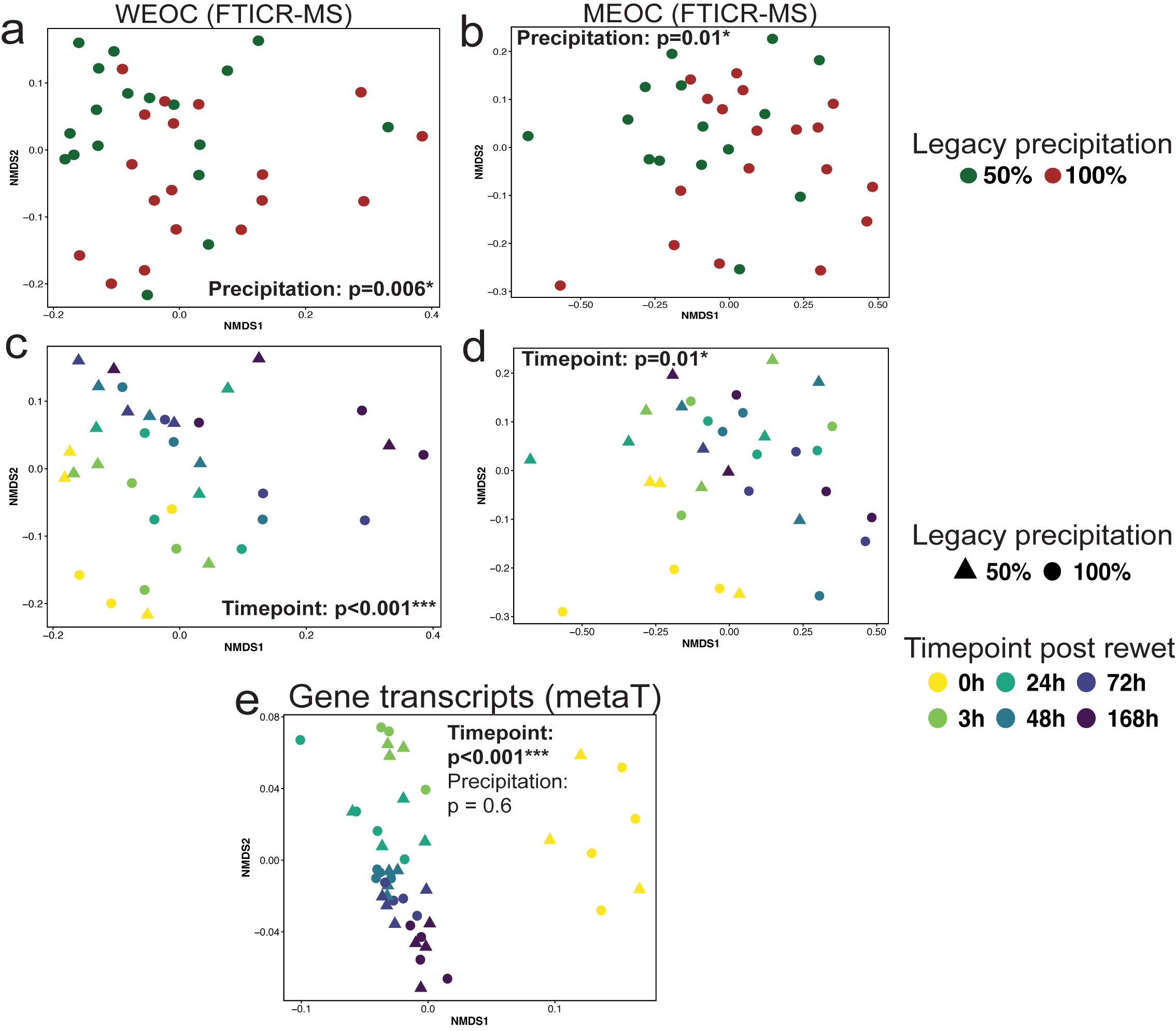

Supplement: Supplementary file 2 — Supplementary Material 1: Figure S1. Shifting patterns in soil organic carbon composition and functional gene transcript abundances during wet-up after summer dry-down for contrasting legacy precipitation regimes. Nonmetric multidimensional scaling (NMDS) ordinations of a) water-extractable organic carbon (WEOC; FTICR-MS), b) methanol-extractable organic carbon (MEOC; FTICR-MS), and e) metatranscriptome transcript abundances of KEGG orthologs (KO) normalized as variance stabilized transformations (VST) of read counts. NMDS of Jaccard distance similarities was performed for MEOC and WEOC (a-d) and of Bray‒Curtis dissimilarity for gene transcripts (e-g). Statistical grouping of treatments was performed using PERMANOVA. *, p < 0.05; **, p < 0.01; ***, p < 0.001. [file 40168_2026_2395_MOESM1_ESM.tif]

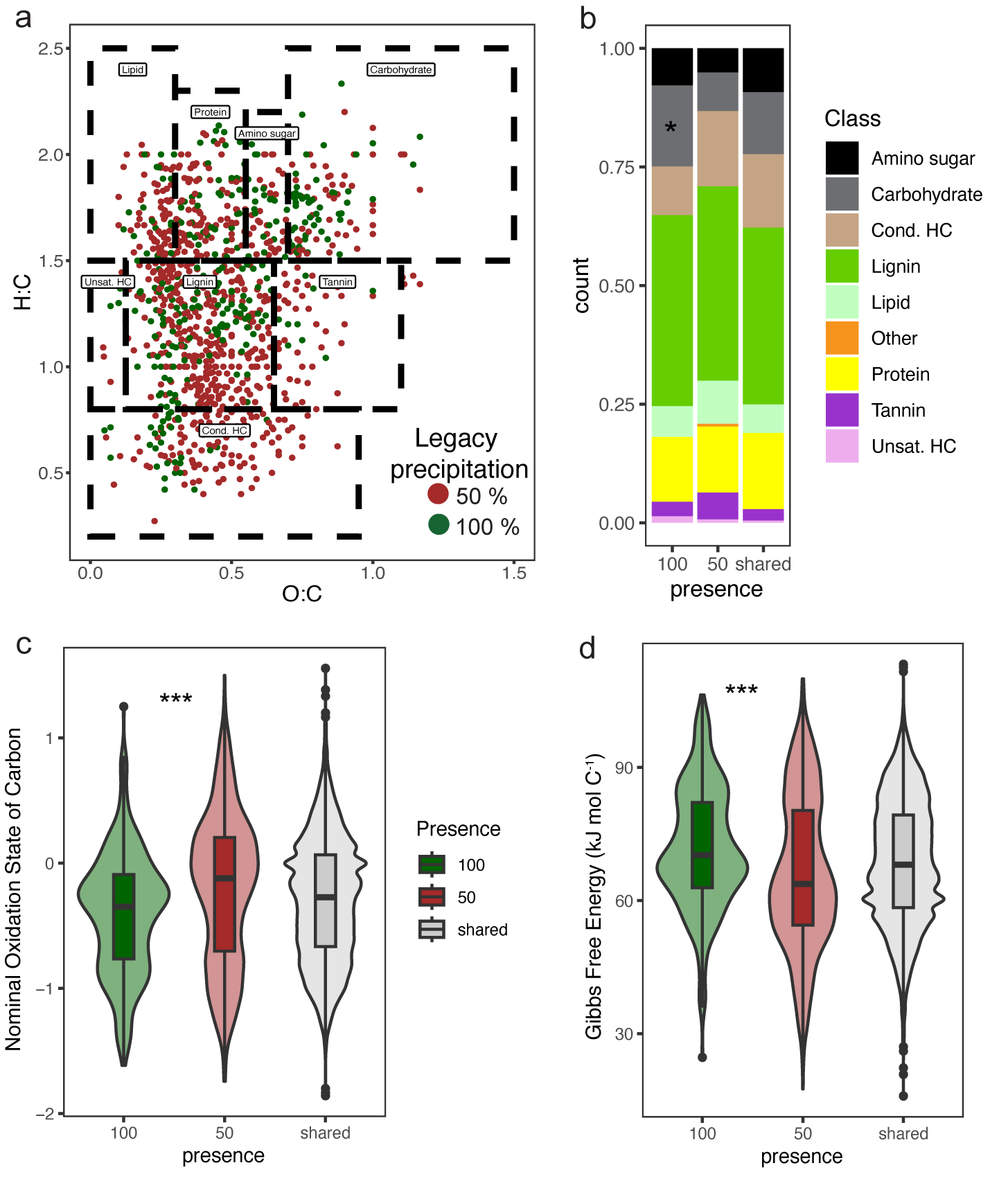

Supplement: Supplementary file 3 — Supplementary Material 2: Figure S2. Legacy precipitation alters the composition and thermodynamics of methanol-extractable organic carbon (MEOC). a) Van Krevelen diagram of compound formula O:C vs H:C ratios, for compounds that were unique to the normal legacy precipitation treatment (100% mean annual precipitation [MAP]) or reduced legacy precipitation treatment (50% MAP). Unique compounds are defined as formulas that were detected in only the 50% or 100% legacy precipitation treatments, where a compound was considered detected if it was present in at least two of the three plots. Dotted boxes mark typical regions for major compound classes (as labeled). b) Relative abundance of compound classes found only in 100%, only in 50%, or in both (shared) legacy precipitation treatments. Asterisks denote classes over-represented in a treatment (G-test, false discovery rate [FDR] corrected). c) Distribution of nominal oxidation state of carbon (NOSC) and d) Gibbs free energy (GFE) for unique and shared compounds within each precipitation treatment. (c-d) Embedded boxplots (box = Q1-Q3, centerline = median, whiskers = min-max excluding outliers [black points]). Legacy precipitation treatment effects for unique compounds were tested with linear mixed-effects models (df = 1). ***, p<0.001. [file 40168_2026_2395_MOESM2_ESM.tif]

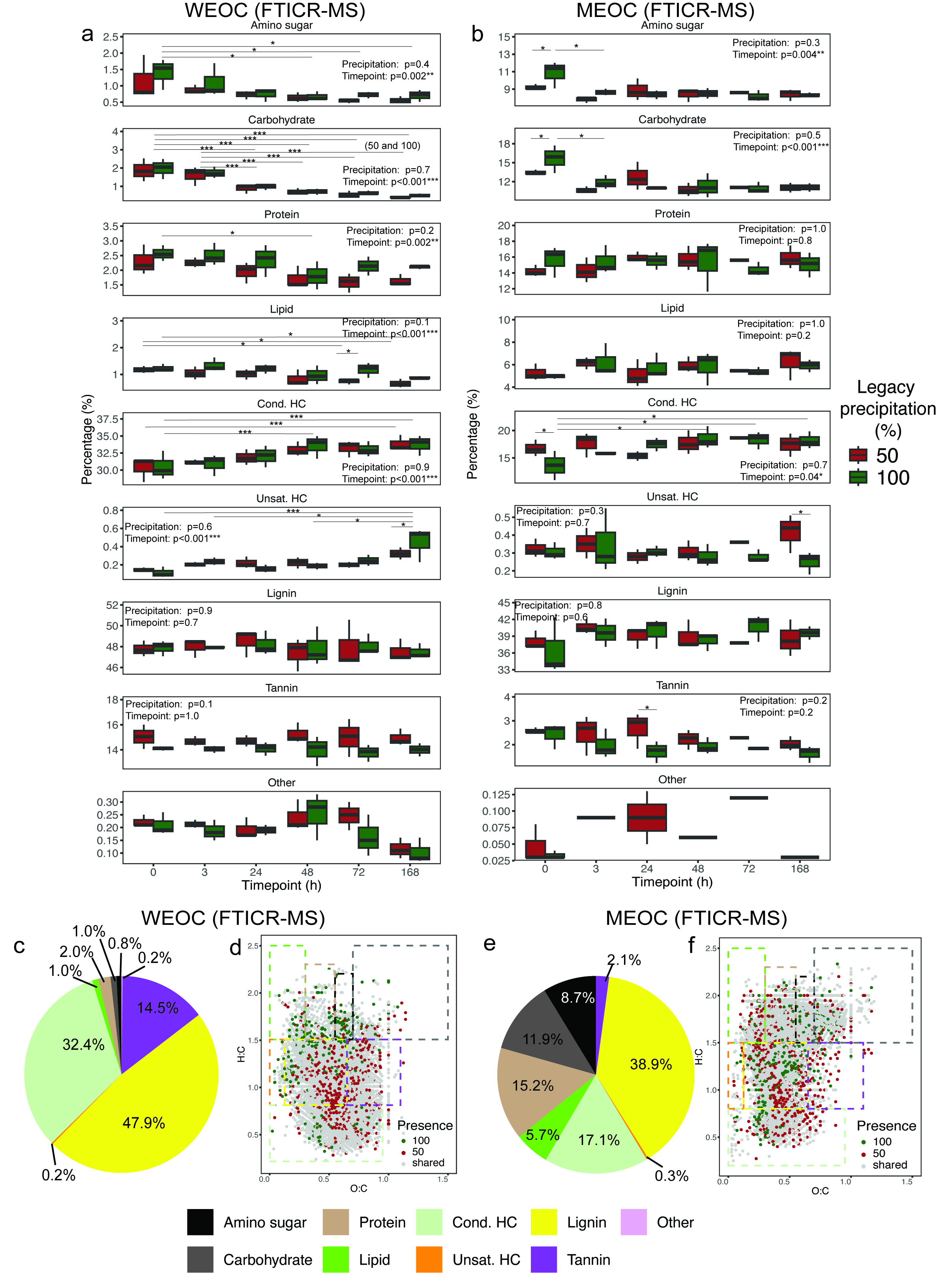

Supplement: Supplementary file 4 — Supplementary Material 3: Figure S3. Composition of water- and methanol-extractable organic carbon (WEOC, MEOC). WEOC and MEOC were measuring using Fourier transform ion cyclotron resonance mass spectrometry (FTICR-MS). Relative abundances of compound classes across time and legacy precipitation treatment for a) WEOC and b) MEOC. Boxplots show Q1-Q3, centerlines= median, and whiskers= min and max values, excluding outliers (black points). Linear mixed-effects model p-values for precipitation (df=1) and timepoint (df=5) are annotated in each plot. Significant pairwise Tukey HSD comparisons are indicated above boxes as lines between comparisons (between precipitation at each timepoint and a given timepoint within each precipitation. (c, e) Pooled class composition (pie charts) across all samples for c) WEOC and e) MEOC. d, f) Van Krevelen diagram of each compound across all samples showing compounds unique to each legacy precipitation treatment and compounds in both (shared) in d) WEOC and f) MEOC. Colors of dotted outlines in Van Krevelen Diagrams indicate compound classes. *, p<0.05; **, p<0.01; ***, p<0.001. [file 40168_2026_2395_MOESM3_ESM.tif]

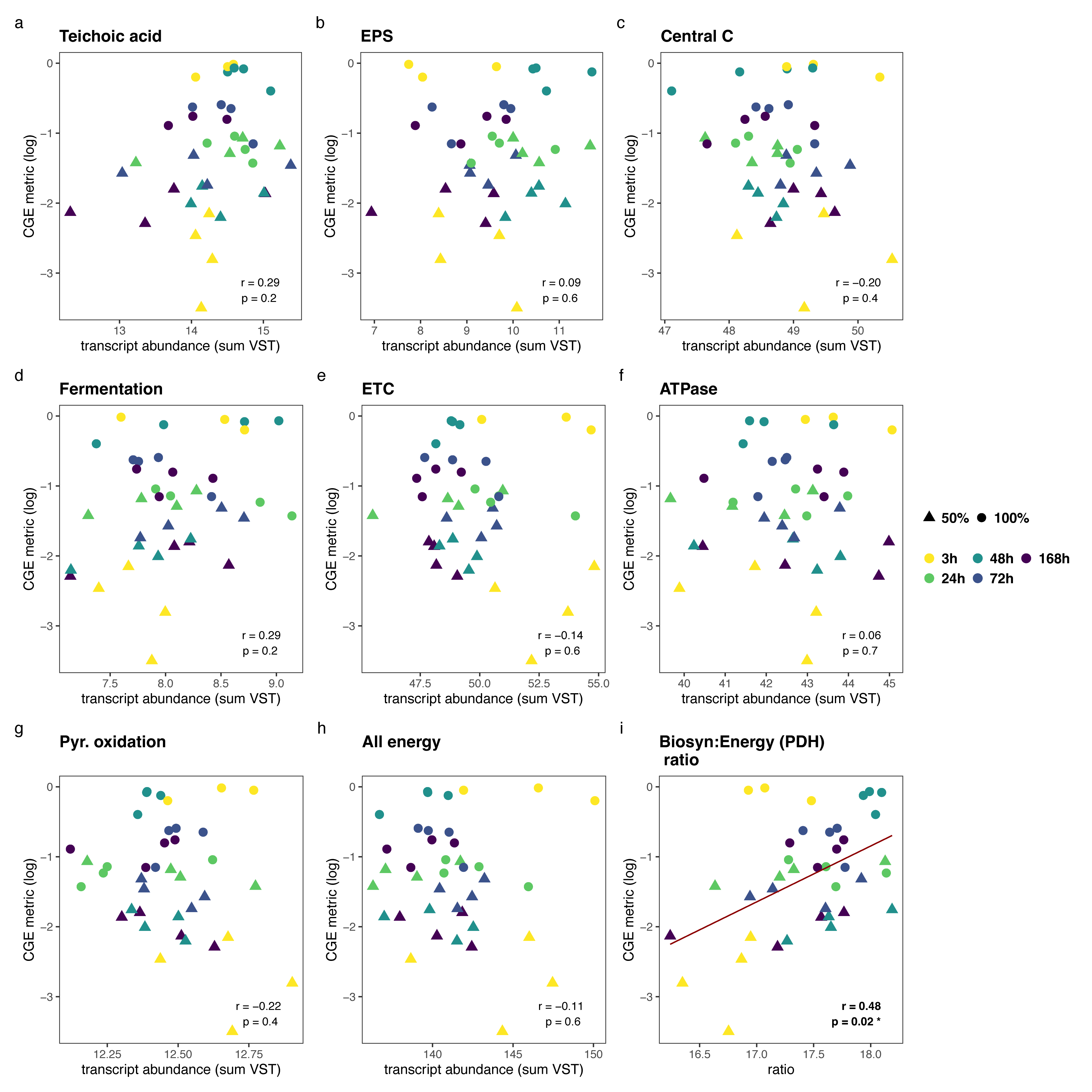

Supplement: Supplementary file 5 — Supplementary Material 4: Figure S4. Relationships between community growth efficiency (CGE) and pathway-level transcript abundances. Pearson correlations were performed between CGE-metric (unitless value between 0 and 1) and summed metatranscriptome transcript abundances (normalized using variance stabilization transformation [VST]). Biosynthesis pathways include a) teichoic acid and b) extracellular polymeric substances (EPS). Energy pathways include c) central C metabolism, d) fermentation, e) electron transport chain (ETC), f) ATPase, and g) pyruvate oxidation. h) Correlation between CGE metric and all energy metabolism genes. d) Correlation between CGE metric and ratio of transcript abundances of biosynthesis: PDH gene, the only CO2-producing energy gene associated with CGE. Red line indicates linear regression line for significant correlations. Pearson correlation coefficient (r) and fdr-corrected p-values labeled on each plot. *, p < 0.05; **, p < 0.01. [file 40168_2026_2395_MOESM4_ESM.tif]

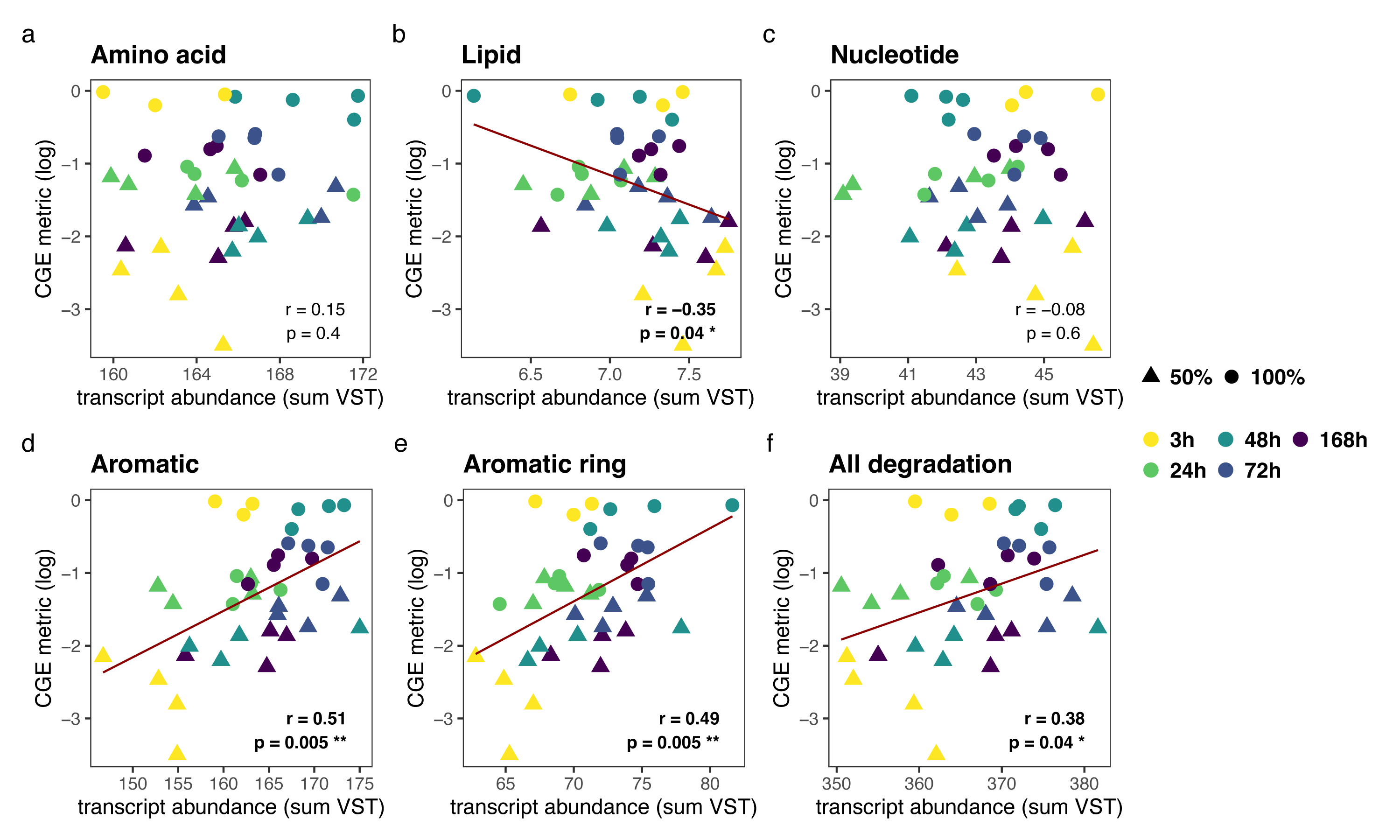

Supplement: Supplementary file 6 — Supplementary Material 5: Figure S5. Relationships between community growth efficiency (CGE) and degradation pathway-level transcript abundances. Pearson correlations were performed between CGE-metric (unitless value between 0 and 1) and summed metatranscriptome transcript abundances (normalized using variance stabilization transformation [VST]). Degradation pathways include a) amino acid (no association), b) lipid (negative association), c) nucleotide (no association), d) aromatic (positive association), and e) aromatic ring degradation (positive association), and f) the sum of all these degradation pathways. Red line indicates linear regression for significant correlations. Pearson correlation coefficient (r) and fdr-corrected p-values labeled on each plot. *, p < 0.05; **, p < 0.01. [file 40168_2026_2395_MOESM5_ESM.tif]

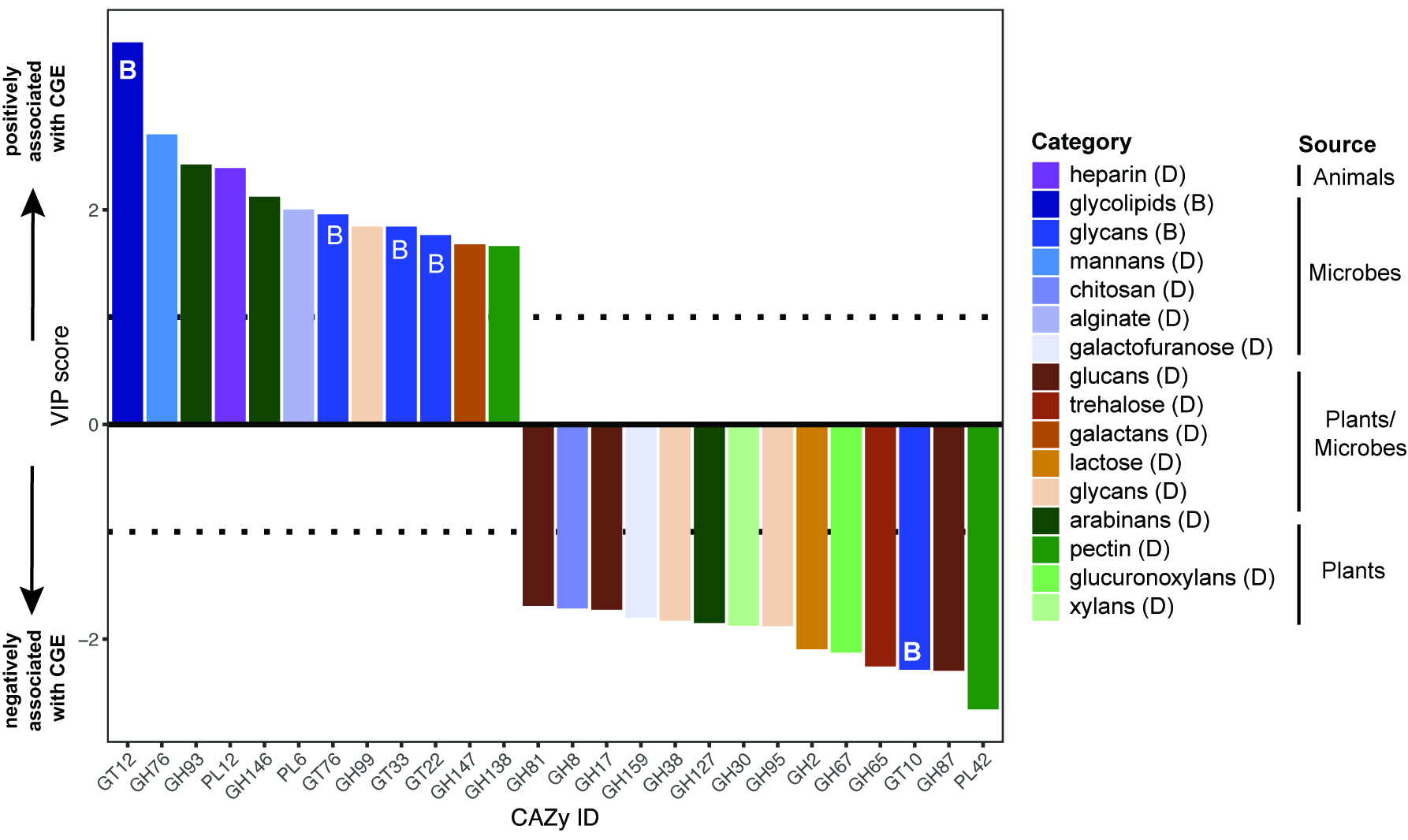

Supplement: Supplementary file 7 — Supplementary Material 6: Figure S6. CGE-associated CAZy genes determined using PLSR. Associations were defined from partial least squares regression (PLSR) with CGE as response variable and metatranscriptome gene transcript abundances as predictor variables (top 10%; VIP > 1.6). The direction of association was determined by the sign of the predictor variable coefficient. VIP scores were adjusted based on the direction of correlation of each CAZy gene with CGE metric. Bars are colored based on the substrate each enzyme degrades, with the exception of the enzymes involved in biosynthesis, as denoted by “B”. Substrate colors indicate sources of substrate (purple, animals; blue, microbes; brown, plants/microbes; green, plants). [file 40168_2026_2395_MOESM6_ESM.tif]

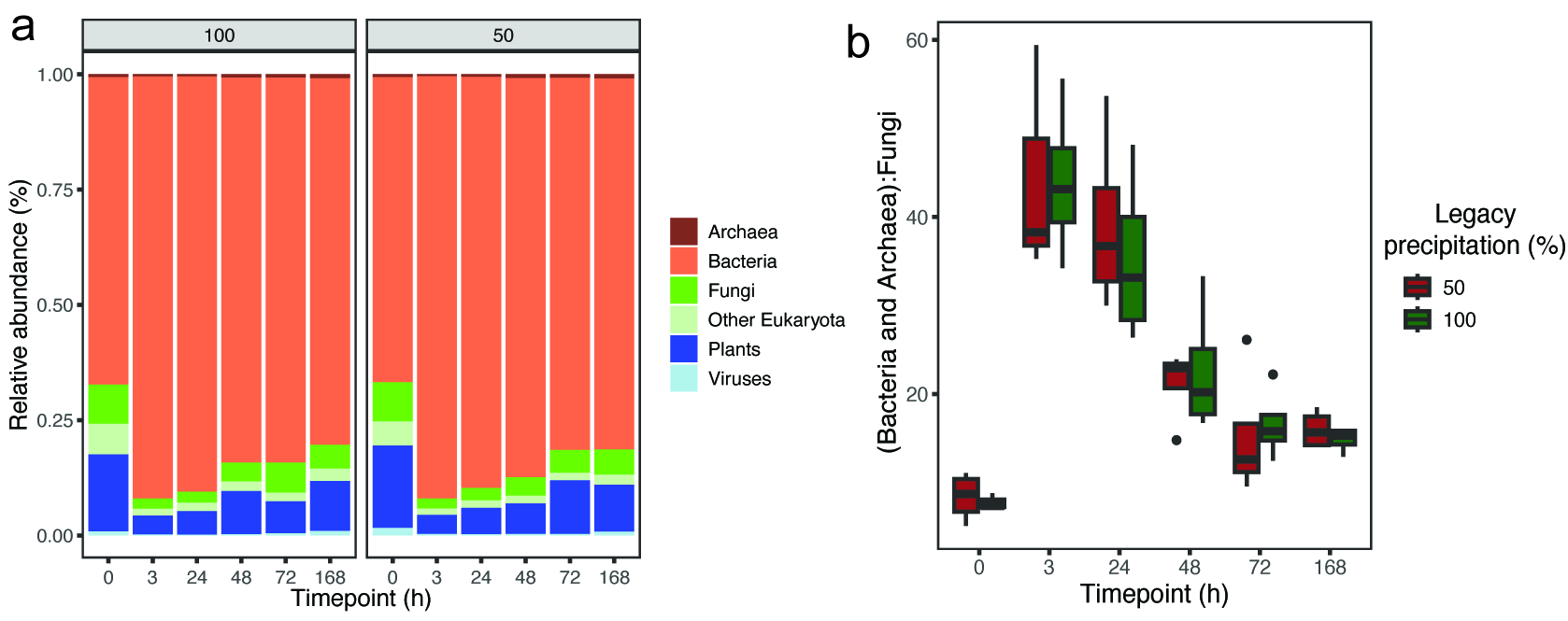

Supplement: Supplementary file 8 — Supplementary Material 7: Figure S7. Taxonomy of metatranscriptomics reads. a) Taxonomic profiles of metatranscriptomics transcript read counts to show abundances of prokaryotes (archaea and bacteria), eukaryotes (fungi, plants, and other), and viruses. b) Ratio of bacteria to fungal read counts across timepoints. [file 40168_2026_2395_MOESM7_ESM.tif]
